# Posterior Reversible Encephalopathy Syndrome in the Immediate Postoperative Period of Gastric Cancer

**DOI:** 10.7759/cureus.49388

**Published:** 2023-11-25

**Authors:** Ankur K Shrivastava, Swatishree Nayak, Narendra Kuber Bodhey, Yamini Patial, Saroj K Pati

**Affiliations:** 1 Ophthalmology, All India Institute of Medical Sciences, Raipur, Raipur, IND; 2 Radiodiagnosis, All India Institute of Medical Sciences, Raipur, Raipur, IND

**Keywords:** immediate postoperative period, chemotherapy, cortical blindness, gastric cancer surgery, posterior reversible encephalopathy syndrome

## Abstract

A 55-year-old female was referred to the Department of Ophthalmology with complaints of bilateral loss of vision. She had undergone subtotal gastrectomy with gastrojejunostomy and lymphadenectomy for poorly differentiated gastric adenocarcinoma in the antropyloric region the day before. On the first postoperative day, she complained of generalised weakness, drowsiness, altered sensorium, and acute, painless, bilateral loss of vision. Ocular examination revealed visual acuity as no perception of light, bilaterally, and normal pupillary light reflexes. Anterior and posterior segment examination was within normal limits. This clinical presentation of altered sensorium and cortical blindness along with characteristic radiological findings (hyperintensity on T2/fluid-attenuated inversion recovery sequence involving the bilateral parieto-occipital lobe extending in asymmetric fashion to the bilateral cerebellum, brainstem, and thalami predominantly involving the white matter with few areas of diffusion restriction on diffusion-weighted imaging sequence predominantly on the left side with gyriform pattern) confirmed the diagnosis of posterior reversible encephalopathy syndrome (PRES). In cancer patients, PRES has been reported in patients on chemotherapy regimen or two weeks after surgery for gastric cancer. Here, we want to draw attention to the fact that PRES may develop in the immediate postoperative period of gastric cancer surgery, as seen in our case.

## Introduction

Posterior reversible encephalopathy syndrome (PRES) is a clinico-radiographic syndrome of diverse aetiologies clustered together with homogeneous features in neuroimaging studies. Initially described as leukoencephalopathy by Hinchey et al. in 1996, this clinical entity was later renamed as PRES [1]. The classic presentation of PRES includes seizures, altered mental status, and changes in vision. Visual disturbances can have varied manifestations ranging from blurred vision to homonymous hemianopia to cortical blindness [1]. Some of the major clinical conditions that predispose to PRES are hypertension, preeclampsia, eclampsia, infection, sepsis, shock, autoimmune diseases, bone marrow or stem cell transplantation, and cancer chemotherapy [1]. Though chemotherapeutic agents used in the treatment of cancer have been identified as an important causative factor, PRES developing in the immediate postoperative period of gastric cancer surgery has rarely been reported. This case report highlights the importance of considering PRES as one of the differential diagnoses of cortical blindness in patients operated for gastric cancer, as this entity can easily be overlooked due to its uncommon occurrence.

## Case presentation

A 55-year-old female was referred to the Department of Ophthalmology with complaints of bilateral loss of vision. She had undergone subtotal gastrectomy with gastrojejunostomy and lymphadenectomy for poorly differentiated gastric adenocarcinoma in the antropyloric region the day before. On the first postoperative day, she complained of generalised weakness, drowsiness, altered sensorium, and acute, painless, bilateral loss of vision. On examination, the patient was afebrile, was obeying commands intermittently, and had slurring of speech, and her pulse rate was 76 beats per minute, sinus rhythm. Her Glasgow Coma Scale (GCS) score was 13 (E4V4M5). Examination of other cranial nerves was normal. Her blood pressure (BP) was recorded as 132/80 mm Hg with a mean arterial pressure (MAP) of 97.3 mm Hg. However, there was an episode of rise in systolic BP up to 180-190 mm Hg (MAP 132.6 mm Hg) in the immediate postoperative period, which persisted over a period of four to five hours.

**Figure 1:**
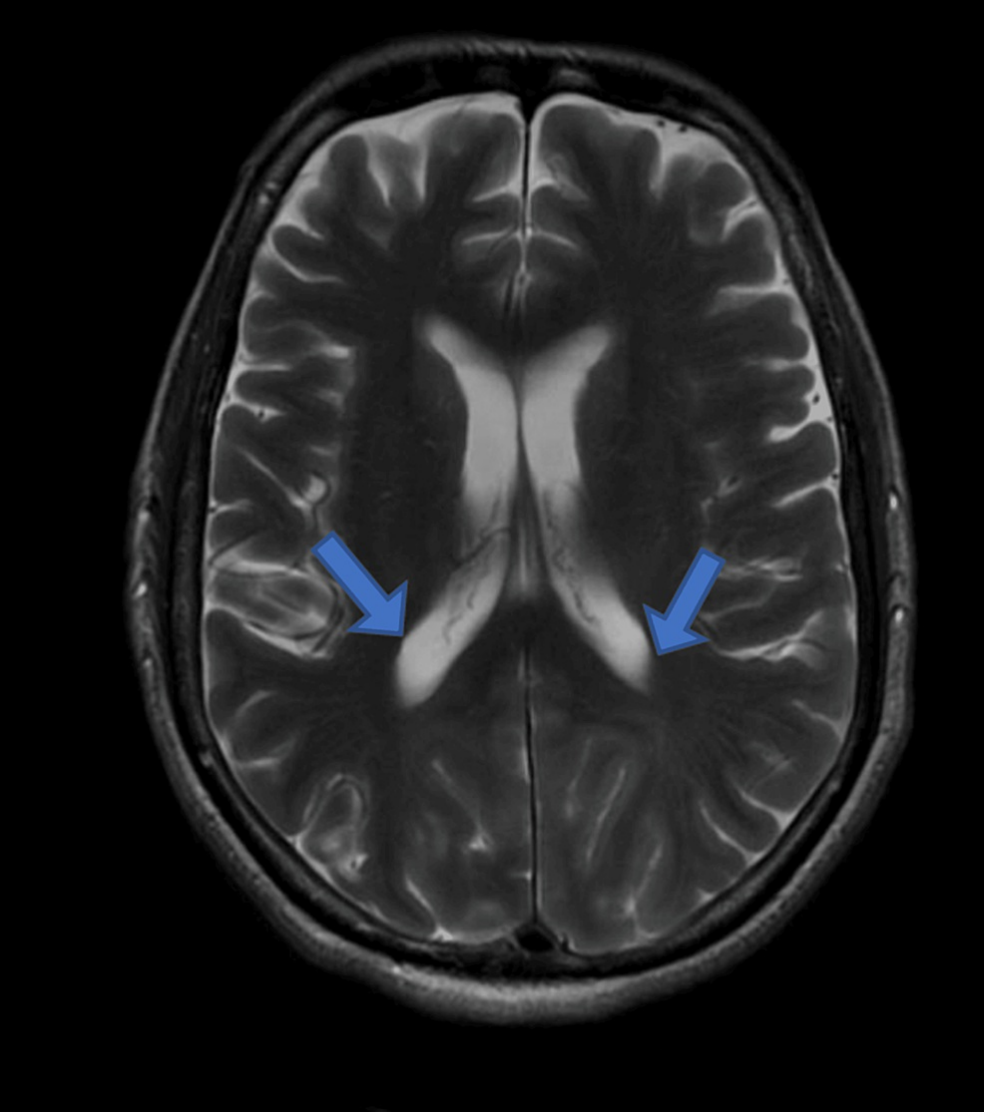
MRI brain coronal section showing hyperintensities in T2-weighted image in the bilateral parieto-occipital lobe extending in asymmetric fashion to the bilateral cerebellum, brainstem, and thalami predominantly involving the white matter

**Figure 2:**
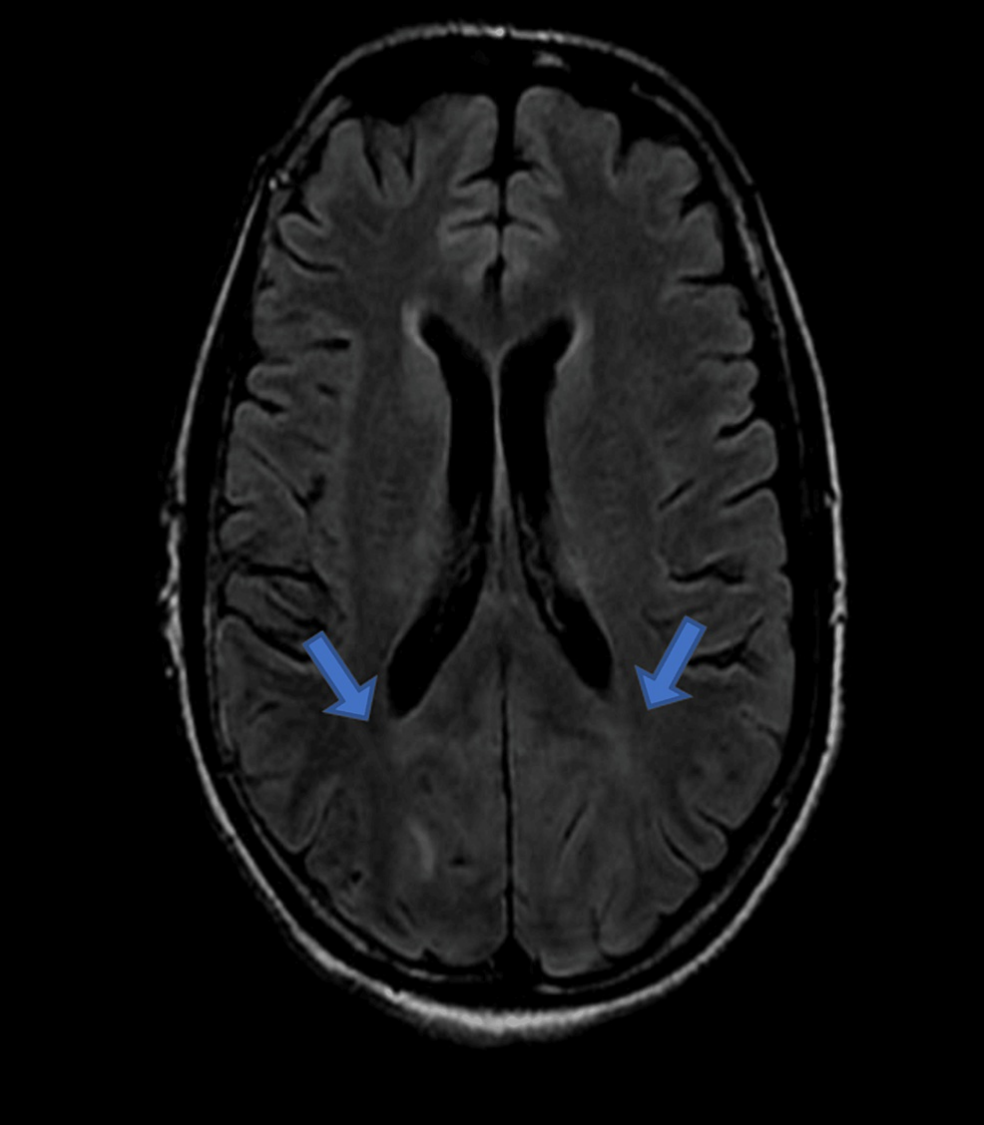
MRI brain coronal section showing hyperintensities in FLAIR sequence in the bilateral parieto-occipital lobe extending in asymmetric fashion to the bilateral cerebellum, brainstem, and thalami predominantly involving the white matter FLAIR: fluid-attenuated inversion recovery

Ocular examination revealed visual acuity as no perception of light, bilaterally. Pupils were 3 mm in size, isocoric, and normally reacting to light. Anterior and posterior segment examination was within normal limits. Based on this clinical presentation, cortical blindness was suspected. It was further confirmed by MRI brain findings which revealed hyperintensity on T2 (Figure 1)/fluid-attenuated inversion recovery (FLAIR) sequence (Figure 2) involving the bilateral parieto-occipital lobe extending in asymmetric fashion to the bilateral cerebellum, brainstem, and thalami predominantly involving the white matter.

**Figure 3:**
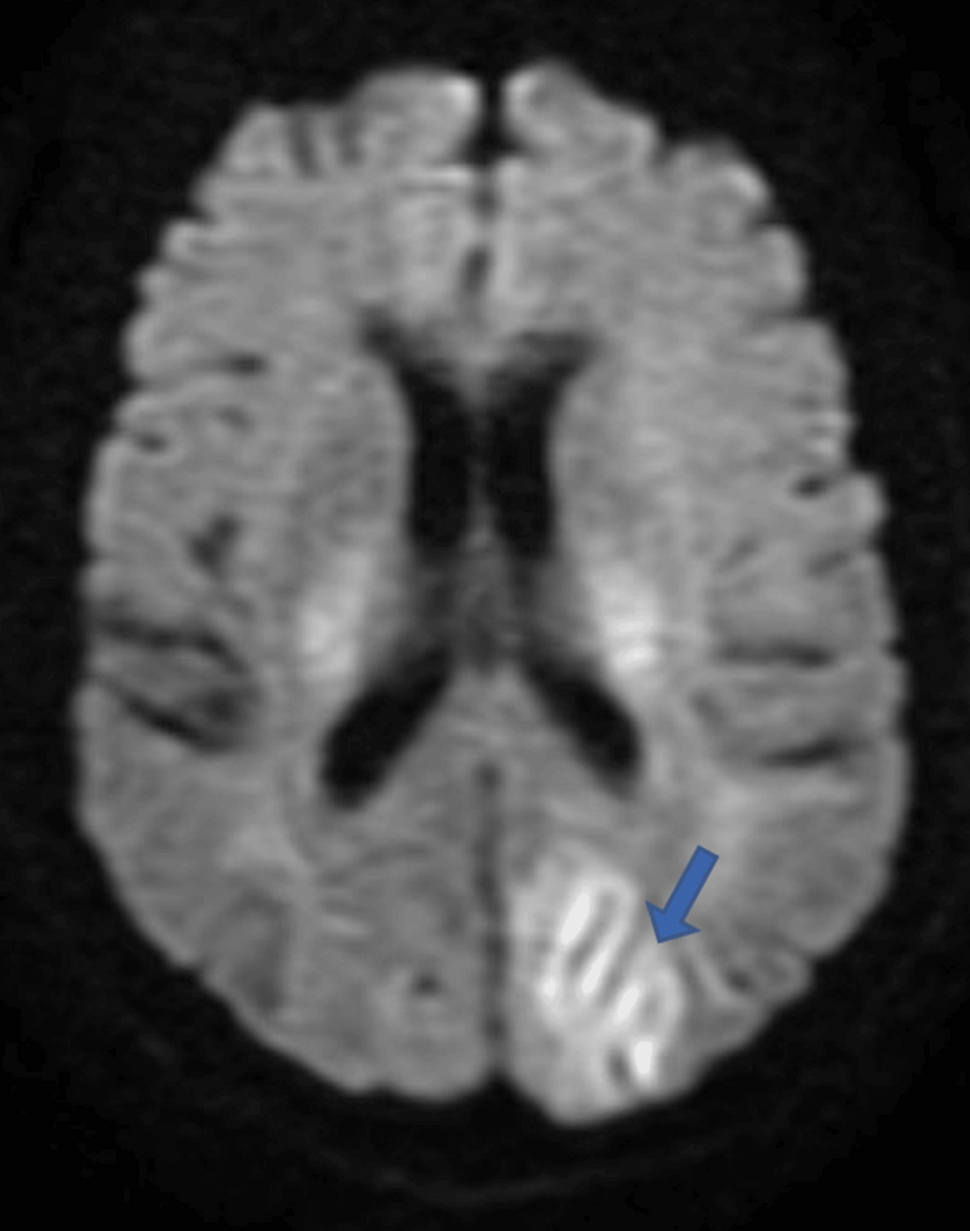
MRI brain showing few areas of diffusion restriction which were of high intensity on DWI sequence predominantly on the left side with gyriform pattern DWI: diffusion-weighted imaging

Few areas of diffusion restriction were seen on diffusion-weighted imaging (DWI) sequence (Figure 3) predominantly on the left side with gyriform pattern.

Signs of sub-acute non-haemorrhagic infarct were seen. However, magnetic resonance (MR) angiography of all major intracranial vessels and carotid Doppler study were normal. The haematological parameters were haemoglobin (Hb) 10.6 g/dl, mean corpuscular haemoglobin (MCH) 20.4 pg, mean corpuscular haemoglobin concentration (MCHC) 30.9 g/dl, and red cell distribution width (RDW) 22.0% which were diagnostic of microcytic hypochromic anaemia. Liver and kidney function tests were normal. Electrocardiography (ECG) revealed sinus rhythm and echocardiography study was normal.

The characteristic radiological findings along with clinical presentation of altered sensorium and bilateral loss of vision confirmed the diagnosis of PRES. Postoperatively, the patient was started on anti-hypertensives along with antibiotics and analgesics. On the ninth postoperative day, the visual acuity improved to counting fingers at 5 m distance in both eyes. The patient was discharged on postoperative day 14, without any neurological deficit and with best corrected visual acuity of 20/40 in both eyes.

## Discussion

Hinchey et al. first described this condition as reversible posterior leukoencephalopathy syndrome in a series of 15 patients [1]. The terminology of this condition has been much debated on ever since its original description in 1996. It was Stott et al. who proposed the term PRES for this condition, and it has been widely recognized [2].

PRES is mostly a disease of middle-aged females with an average age of presentation being close to 40 years [3-4]. A consistent association of PRES has been shown with clinical conditions like hypertensive encephalopathy, eclampsia, renal failure, autoimmune diseases, sepsis, and shock [5]. PRES is progressively being more identified in cancer patients on multidrug chemotherapy regimens, monoclonal antibodies, immunosuppressants, and solid organ and stem cell transplantation and more so in hematopoietic malignancies [6-7]. There have also been some case reports of PRES associated with blood transfusion to correct severe anaemia in cancer patients [8].

The clinical manifestations of PRES can have a wide array ranging from severe headache, altered mental status with disorientation, changes in the level of consciousness, generalised seizures to visual complaints [9]. The innate absence of adrenergic innervation in the vertebrobasilar system results in oedematous changes in the parieto-occipital lobes accounting for visual complaints [10]. Though cortical blindness is the most common visual abnormality, homonymous hemianopia, visual neglect, impairment of facial recognition (prosopagnosia), denial of blindness (Anton's syndrome), visual hallucinations, and blurred vision can also occur [9,11]. There have also been reports of some rare visual manifestations like simultanagnosia due to dorsal stream dysfunction and achromatopsia due to lesion in area V4 of the occipital cortex [12,13]. Visual complaints are a more common finding in eclampsia-related PRES (50%), while PRES with other aetiologies present with visual disturbances in only 27.8% of cases [14].

The pathophysiology of PRES remains a matter of debate. The proposed mechanisms are 1) failure of cerebral autoregulation and endothelial cell dysfunction leading to vasogenic oedema and 2) vasospasm leading to ischaemia and cytotoxic oedema [15]. Although hypertension is commonly associated with PRES, it has also been reported to occur in 15-20% of normotensive or hypotensive cases. In such cases, it is the fluctuation of BP rather than the acute rise in BP which has been postulated for the development of PRES [10].

**Figure 4:**
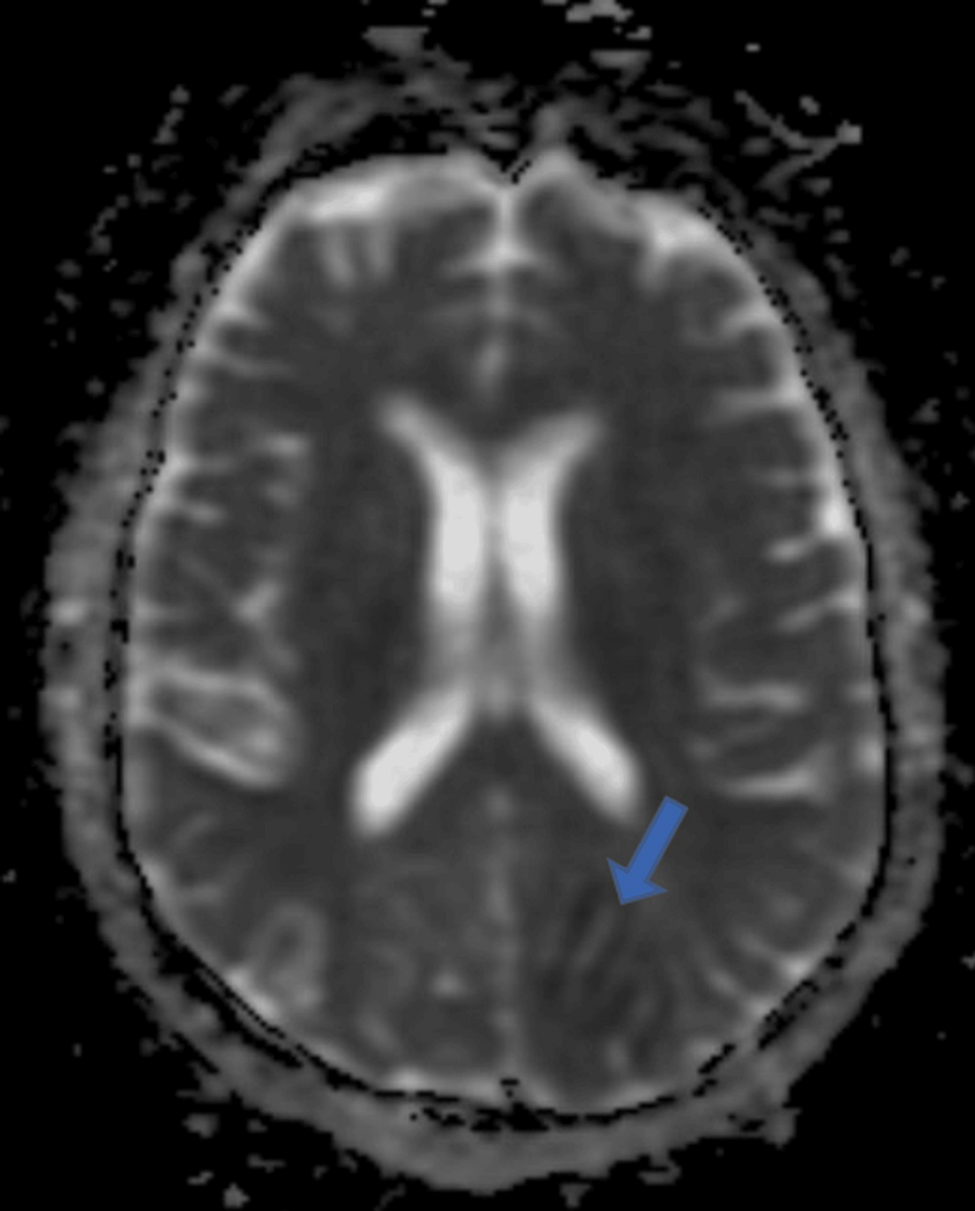
MRI brain showing few areas of diffusion restriction which were of low intensity on the ADC map on the left side with gyriform pattern ADC: apparent diffusion coefficient

Our patient, a 55-year-old female, a postoperative case of gastric cancer, presented with features of altered sensorium and bilateral, sudden loss of vision on the first postoperative day. The clinical and radiological features confirm the diagnosis of PRES. Areas of diffusion restriction which were of high intensity on DWI (Figure 3) and low intensity on the apparent diffusion coefficient (ADC) map (Figure 4) were seen, thus implying "vasospasm leading to ischaemia and cytotoxic oedema theory" as the causative mechanism in our case.

Preoperative records reveal BP to be within normal limits. However, there was an episode of acute rise in systolic BP up to 180-190 mm Hg in the immediate postoperative period, which persisted over a period of four to five hours. Inadequate pain management was the most probable cause of this wide fluctuation in BP. This fluctuation in BP could have led to vasospasm resulting in hypoperfusion, ischaemia, and finally cytotoxic oedema and infarct, as found on the MRI in this case. The posterior region of the brain is more susceptible to such fluctuation because of the little sympathetic innervation in the posterior fossa [10].

Singer et al. in their study on PRES in adult cancer patients stated chemotherapy as the potential risk factor [14]. Of the 31 cases studied, only three had gastric cancer as aetiology and were on chemotherapy regimen. Similar findings were reported by Kamiya-Matsuoka et al. [16]. Immune response to unique tumour antigens and the direct effect of chemotherapy on endothelial cells are considered as the underlying mechanisms of PRES in cancer chemotherapy patients [15]. In our patient, PRES developed even before the initiation of chemotherapy.

In a case reported by Higashi et al., symptoms of blurred vision and PRES developed two weeks after surgery for early gastric cancer [17]. The patient, though was normotensive, had scleroderma which contributed to hypertension reported in the postoperative period. However, it took six months for visual acuity to recover, and there was residual visual field defect too. Here, we want to draw attention to the fact that PRES may develop in the immediate postoperative period of gastric cancer surgery, as seen in our case. Since ophthalmologists would be part of the team where such cases are referred to, they should be vigilant about PRES as a differential diagnosis of cortical blindness in the immediate postoperative period of cancer surgery. Prompt diagnosis and early treatment of underlying cause can help reduce the incidence of permanent residual deficits.

## Conclusions

PRES has been reported in cancer patients on a chemotherapy regimen or two weeks after gastric cancer surgery. We report a case where PRES has developed in the immediate postoperative period even without the initiation of anti-neoplastic medications.
