# Correction: Posterior Reversible Encephalopathy Syndrome in the Immediate Postoperative Period of Gastric Cancer

**DOI:** 10.7759/cureus.c153

**Published:** 2024-01-18

**Authors:** Ankur K Shrivastava, Swatishree Nayak, Narendra Kuber Bodhey, Yamini Patial, Saroj K Pati

**Affiliations:** 1 Ophthalmology, All India Institute of Medical Sciences, Raipur, Raipur, IND; 2 Radiodiagnosis, All India Institute of Medical Sciences, Raipur, Raipur, IND

This article's author list has been corrected to include Dr. Swatishree Nayak as second author. Dr. Nayak was mistakenly excluded from the original submission and the authors requested this addition. After careful review, the journal has approved this correction and Dr. Nayak has been added to the article as second author.

